# Endophytic bacteria associated with turmeric, ginger and white radish exhibit dual roles as producers of antibacterial metabolites and carriers of multidrug resistance

**DOI:** 10.1038/s41598-026-64927-5

**Published:** 2026-08-31

**Authors:** Sudipta Saha, A. K. M. Shohanur Rahman, Md. Zahirul Islam, Md. Ajijur Rahman

**Affiliations:** 1 Departemnt of Pharmacy, University of Rajshahi, Rajshahi, 6205 Bangladesh; 2 Departemnt of Pharmacy, Eastern Unievrsity, Dhaka, 1345 Bangladesh; 3 Departemnt of Pharmacy, East West University, Dhaka, 1212 Bangladesh

**Keywords:** Endophytic bacteria, Antimicrobial resistance, Multidrug-resistant bacteria, Edible rhizomes, *Zingiber officinale*, *Curcuma longa*, *Raphanus sativus*, Antibacterial activity, Food safety, One Health, *Burkholderia contaminans*, *Enterobacter cloacae*, Biotechnology, Microbiology

## Abstract

The increasing global burden of antimicrobial resistance (AMR) necessitates the exploration of unconventional microbial niches for novel bioactive compounds. Simultaneously, it is also necessary to evaluate their role in the dissemination of resistant bacteria through the food chain. Endophytic bacteria inhabiting edible plant tissues represent a largely underexplored interface between environmental microbiota and human exposure. In this exploratory study, endophytic bacteria were isolated from fresh rhizomes of *Zingiber officinale* (ginger), *Curcuma longa* (turmeric), and *Raphanus sativus* (white radish), and characterized for their antimicrobial activity and antibiotic resistance profiles. A total of 24 endophytic bacterial isolates were isolated from fresh rhizomes of turmeric, ginger and white radish using tissue segmentation method. Among them, eight isolates (33.33%) exhibited weak to strong inhibitory activity against multidrug-resistant clinical pathogens, including *Escherichia coli*, *Escherichia fergusonii*,* Shigella spp.*, *Staphylococcus aureus*, and *Bacillus cereus*. Molecular identification based on 16S rRNA gene sequencing revealed that the isolate RD-1(b) was affiliated with *Burkholderia contaminans* and TM-4(b) and RD-2(c) with *Enterobacter cloacae*. Crude extracellular metabolites extracted from the RD-1(b) retained broad-spectrum antibacterial activity. Notably, these isolates demonstrated resistance to multiple antibiotics, including β-lactams, macrolides, and tetracyclines, while remaining susceptible to selected agents such as ciprofloxacin and cotrimoxazole. The coexistence of antimicrobial activity and multidrug resistance within endophytic bacteria highlights their dual role as both potential sources of bioactive metabolites and reservoirs of AMR determinants. Given that these rhizomes are commonly consumed raw or minimally processed, the presence of such bacteria potentially raises food safety concerns. The possible beneficial role of these endophytes should also be explored.

**Supplementary Information:**

The online version contains supplementary material available at https://doi.org/10.1038/s41598-026-64927-5.

## Introduction

Antimicrobial resistance (AMR) has emerged as one of the most pressing global public health challenges, undermining the efficacy of existing antimicrobial therapies and complicating the treatment of infectious diseases. Recent global estimates indicate that AMR is directly responsible for approximately 1.27 million deaths annually and contributes to nearly 5 million deaths worldwide, with a disproportionate burden in low- and middle-income countries^1^. Although, the misuse and overuse of antibiotics are considered the major drivers of selction and evolution of AMR, increasing evidence highlights the critical role of environmental and food-associated microbial reservoirs in the evolution and dissemination of resistance^2,3^.

Plants harbour complex and dynamic microbial communities, including endophytes. Endophytes are microorganisms that colonize internal plant tissues without causing apparent disease^4^. Endophytic bacteria establish close interactions with their host plants and are recognized as prolific producers of structurally diverse secondary metabolites with many medicinal properties including antimicrobial, antifungal, and anticancer properties^4–6^.

Historically, natural products derived from soil-dwelling bacteria particularly *Streptomyces* spp. have been a cornerstone of antibiotic discovery^7,8^; however, frequent rediscovery of known compounds from microorganisms isolated from conventional sources have been common in recent years^9^, which necessitates the investigation of alternative ecological niches such as plant endospheres^10–12^.

In parallel with their biotechnological potential, endophytic bacteria may also harbor intrinsic or acquired AMR genes, reflecting their adaptation to competitive microbial environments and exposure to antibiotics^13^. This duality raises important concerns regarding their role as reservoirs and vectors of AMR within the food chain^3,13,14^. Edible vegetables and other plants, particularly those consumed raw or with minimal processing, represent a direct route for the transfer of associated microbiota including potential AMR carrying opportunistic pathogens to humans^14^. Fresh vegetables and herbs have previously been implicated in the transmission of antibiotic-resistant bacteria, highlighting the need for comprehensive microbiological assessment of plant-associated microbial communities^15,16^.

Rhizomatous plants such as ginger (*Zingiber officinale*), turmeric (*Curcuma longa*), and white radish (*Raphanus sativus*) are widely consumed in both culinary and traditional medicinal contexts, often in raw or minimally processed forms. Despite their widespread dietary use and recognized health benefits, limited attention has been given to the characterization of their internal microbiota, particularly in relation to antimicrobial resistance and potential source of antibiotics.

Previous studies have demonstrated that endophytes isolated from medicinal plants can exhibit significant antimicrobial activity against clinically relevant pathogens, suggesting their potential role in natural defense mechanisms and as sources of novel therapeutics^5,10,11^. Previous studies on endophytes of turmeric, ginger and white radish focused on the growth promoting activity^17–19^. However, the coexistence of antimicrobial production and antibiotic resistance within endophytic populations remains insufficiently explored.

Unlike previous studies that primarily investigated plant growth promotion and antimicrobial activity of endophytes associated with the rhizomes of ginger, turmeric, and white radish, the present exploratory study simulatneously examined the antibacterial activity of the isolates against multidrug-resistant clinical pathogens and antibiotic resistance profiles of the same endophytic isolates, thereby highlighting their dual role within a One Health framework. According to our knowledge, this is the first study investigating edible rhizome-associated endophytic bacteria from Bangladesh from both antimicrobial discovery and antimicrobial resistance perspectives.

## Materials and methods

### Sample collection

Fresh, healthy rhizomes of *Zingiber officinale* (ginger), *Curcuma longa* (turmeric), and *Raphanus sativus* (white radish) were collected from agricultural fields in Naogaon, Bangladesh. Samples were transported to the laboratory in sterile polyethylene bags and processed within 24 h. Prior to processing, rhizomes were washed under running tap water to remove adhering soil and debris.

### Surface sterilization and validation

Rhizome segments (~ 1 cm²) were surface sterilized following a modified protocol^20^. Samples were sequentially immersed in 70% ethanol (3 min), 0.4% HgCl₂ (1 min), and 70% ethanol (2 min), followed by three rinses with sterile distilled water (1 min each).

To confirm the effectiveness of surface sterilization, aliquots of the final rinse water were plated onto starch-casein-nitrate agar (SCNA), and sterilized tissue imprints were also placed on agar plates. Plates were incubated at 30 ± 2 °C for 5–7 days. The absence of microbial growth was considered indicative of successful surface sterilization.

### Isolation of endophytic bacteria

Surface-sterilized tissues were aseptically dissected to obtain inner segments (~ 0.5 cm), which were placed onto starch–casein–nitrate agar (SCNA) plates supplemented with nystatin (60 µg/mL) and potassium dichromate (K₂Cr₂O₇; 60 µg/mL) to suppress fungal and fast-growing bacterial contaminants^21^. Plates were incubated at 30 ± 2 °C for up to 14 days and monitored daily. Emerging colonies were subcultured repeatedly to obtain pure isolates. Pure cultures were maintained on yeast extract–glucose agar (YEGA) at 4 °C and in 20% glycerol stock in freezers.

### Screening of antibacterial activity

Antibacterial activity was assessed using the cross-streak method ^8^. Each isolate was streaked centrally on YEGA plates and incubated at 37 °C for 48 h. Subsequently, test organisms were streaked perpendicular to the producer strain. Test pathogens included multidrug-resistant strains of *Escherichia coli*, *Shigella flexneri*, *Shigella boydii*, *Escherichia fergusonii*, *Staphylococcus aureus*, and *Bacillus cereus*. Bacterial suspensions were standardized to 0.5 McFarland turbidity. Inhibition was measured as the distance (mm) between the producer strain and the test organism.

### Production and extraction of crude antibacterial metabolites

To verify that the antibacterial activity observed during preliminary screening was attributable to diffusible extracellular metabolites, the most bioactive isolate, RD-1(b), was selected for metabolite extraction. Preliminary optimization experiments identified yeast extract glucose agar (YEGA) and an incubation period of 48 h as the most suitable conditions for antibacterial metabolite production; therefore, these conditions were used for subsequent extraction.

RD-1(b) was cultivated on YEGA plates at 37 °C for 48 h. Following incubation, bacterial biomass was carefully removed from the agar surface using a sterile glass slide, and the agar medium was cut into small pieces. An equal volume of ethyl acetate (1:1, v/v) was added, and the mixture was extracted overnight at room temperature with gentle agitation. The extract was centrifuged at 6,000 rpm for 10 min, after which the organic phase was collected and evaporated to dryness at room temperature.

The dried crude extract was dissolved in ethyl acetate to obtain a final concentration of 5 mg/mL. Sterile paper discs were impregnated with 60 µL of the extract (300 µg/disc), air dried under aseptic conditions, and evaluated for antibacterial activity against representative multidrug-resistant clinical isolates using the Kirby–Bauer disc diffusion method. Kanamycin (30 µg/disc) and ethyl acetate served as positive and negative controls, respectively.

### DNA extraction and 16S rRNA gene sequencing

Genomic DNA was extracted using the phenol–chloroform method. The 16S rRNA gene was amplified using universal primers 27F and 1492R in a 25 µL reaction mixture containing template DNA, primers, MgCl₂, and Taq Master Mix. PCR conditions were: initial denaturation at 95 °C for 5 min; 35 cycles of 94 °C for 30 s, 54 °C for 30 s, and 72 °C for 45 s; followed by final extension at 72 °C for 10 min. PCR products were visualized on 1% agarose gel, purified, and sequenced using Sanger sequencing. The 16S rDNA sequences of three isolated endophytes – TM-4(b), RD-2(c) and RD-1(b) – were submitted to GenBank and the accession numbers of PZ424636, PZ424637 and PZ424638 were assigned, respectively.

### Phylogenetic analysis

Sequences were compared with reference sequences in the NCBI GenBank database using EZBioCloud. Multiple sequence alignment was performed using ClustalW. The evolutionary history was inferred by using the Maximum Likelihood method and Kimura 2-parameter model^22^. The bootstrap consensus tree inferred from 50 replicates is taken to represent the evolutionary history of the taxa analyzed^23^. Branches corresponding to partitions reproduced in less than 50% bootstrap replicates are collapsed. The percentage of replicate trees in which the associated taxa clustered together in the bootstrap test (50 replicates) are shown next to the branches^23^. There were a total of 1479 positions in the final dataset. Evolutionary analyses were conducted in MEGA X^24^. The 16S rDNA sequence of *E. coli* ATCC 11775 was used as an outgroup.

### Morphological and physiological characterization

Colony morphology was recorded after incubation on YEGA at 37 °C for 48 h. Gram staining was performed to determine cell morphology and Gram reaction. Temperature tolerance was assessed at 25, 30, 37, 42, 45, and 50 °C, with growth recorded qualitatively after 48 h. Metabolic profiling was conducted using YEGA supplemented with individual carbon sources (glucose, lactose, sucrose, xylose, fructose, and maltose). Plates were incubated at 37 °C for 48 h, and growth was categorized as poor (+), moderate (++), or abundant (+++).

### Antibiotic susceptibility testing

Antibiotic susceptibility profiles of selected isolates were determined using the Kirby–Bauer disk diffusion method on Mueller–Hinton agar^25^. Bacterial suspensions (0.5 McFarland) were spread uniformly onto plates. The following antibiotic discs were used: ciprofloxacin (5 µg), cotrimoxazole (25 µg), azithromycin (15 µg), penicillin-G (10 IU), tetracycline (10 µg), nitrofurantoin (300 µg), gentamicin (10 µg), and ceftriaxone (30 µg). Plates were incubated at 37 °C for 24 h, and inhibition zones were measured in millimeters. Results were interpreted according to Clinical and Laboratory Standards Institute (CLSI) guidelines (2018).

### Statistical analysis

All experiments were performed in triplicate, and results are presented as mean values where applicable. Data were analyzed descriptively due to the exploratory nature of the study.

## Results

### Isolation and recovery of endophytic bacteria from edible rhizomes

**Fig. 1:**
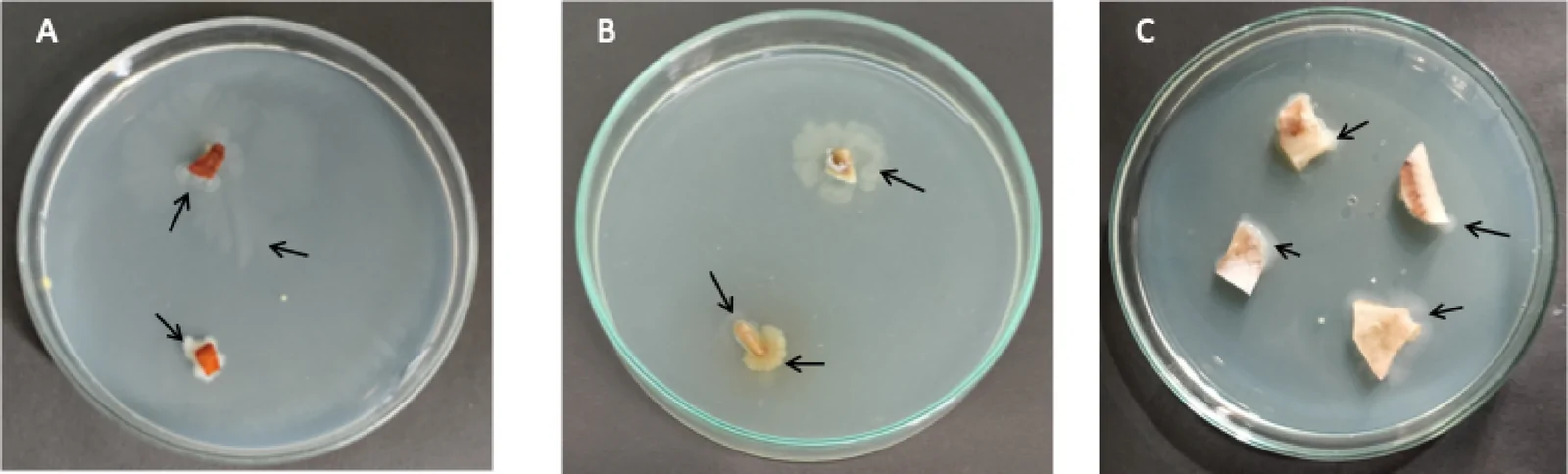
Isolation of endophytic bacteria from fresh rhizomes of turmeric (**A**), ginger (**B**) and white radish (**C**) using tissue segmentation method using SCNA media supplemented with nystatin and K_2_Cr_2_O_7_. Endophyte bacteria appeared to grow after 7th day from the side of the tissues. The colonies are marked with black arrows. Plates were incubated at 30–32 °C for about two weeks. Only one representative plate of each type of source is shown.

**Fig. 2:**
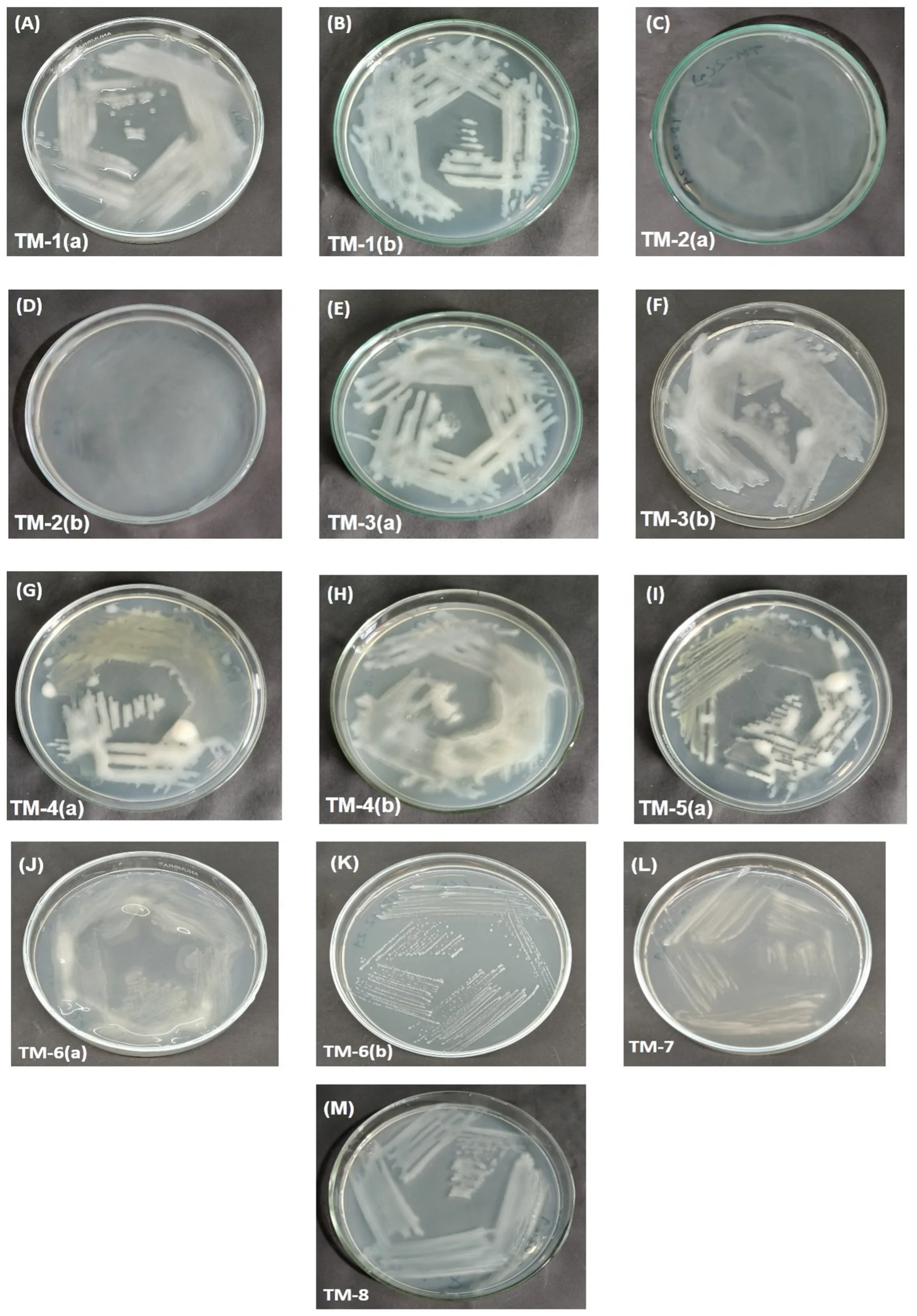
Morphology of the pure culture of endophytes isolated from fresh rhizome of turmeric on yeast-extract glucose agar (YEGA) media (**A**-**M**). The numbers on the bottom-left of each plate are the strain numbers used for the isolates in this study.

**Fig. 3:**
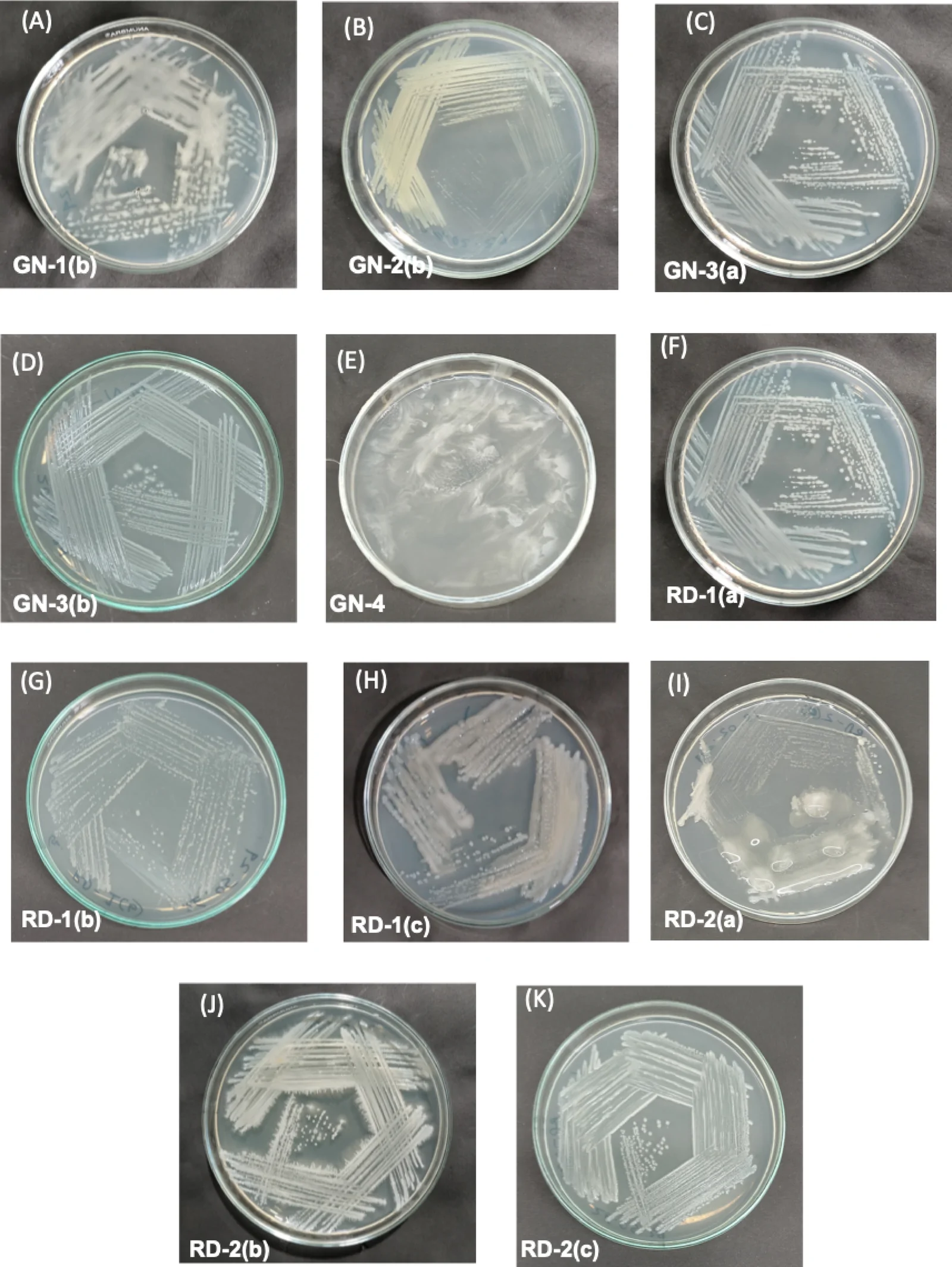
Morphology of the pure culture of endophytes isolated from fresh rhizomes of ginger (**A**-**E**) and white radish (**F**-**K**) on yeast-extract glucose agar media (**A**-**M**). The numbers on the bottom-left of each plate are the strain numbers used in this study.

Endophytic bacteria were successfully isolated from the inner tissues of *Curcuma longa* (turmeric), *Zingiber officinale* (ginger), and *Raphanus sativus* (white radish) following surface sterilization and selective culturing of inner tissue segments on SCNA medium (Fig. 1). A total of 24 morphologically distinct bacterial isolates were isolated in pure culture, comprising 13 isolates from turmeric, 5 from ginger, and 6 from white radish (Fig. 2 and Fig. 3).

Bacterial emergence from internal tissues was observed after approximately 7–8 days of incubation, confirming successful suppression of epiphytic contaminants and selective recovery of endophytes.

### Screening of antibacterial activity of the isolates against multidrug-resistant pathogens

**Fig. 4:**
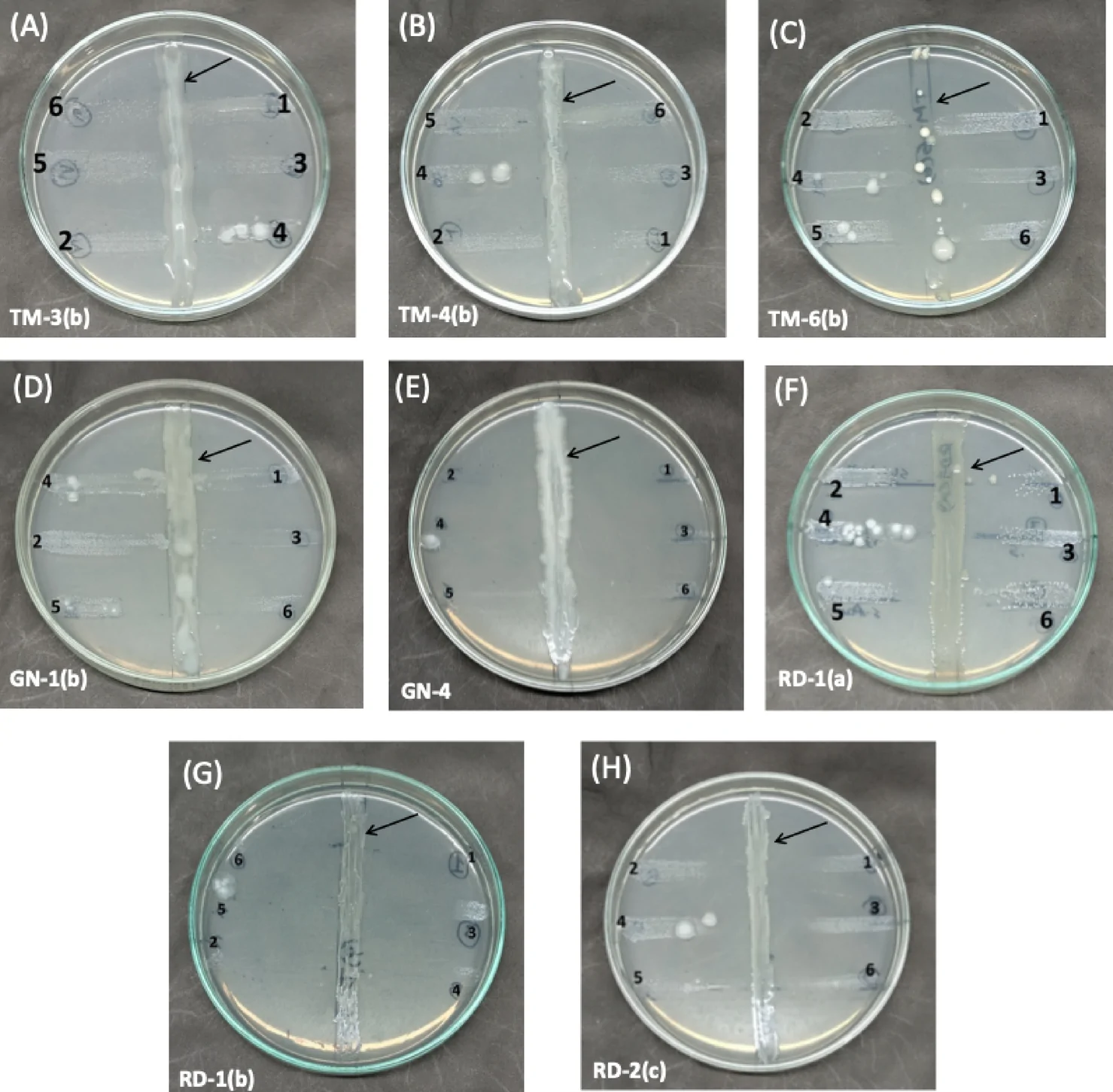
Antibacterial activity of selected turmeric (**A**-**C**), ginger (**D**-**E**) and radish (**F**-**H**) isolates against a wide range of drug-resistant test bacteria. The black arrows show locations of the single line streaking of the endophyte isolates. After streaking on a single line vertically, the plates were incubated for 48 h followed by inoculation of test bacteria perpendicularly and incubated for 16–24 h to see inhibitory activity. The numbers on two sides on the plates indicate the inoculated test bacteria, where *S. flexneri (1)*,* S. boydii (2)*,* E. fergusonii (3)*,* B. cereus (4)*,* S. aureus (5) and E. coli (6).*

**Fig. 5:**
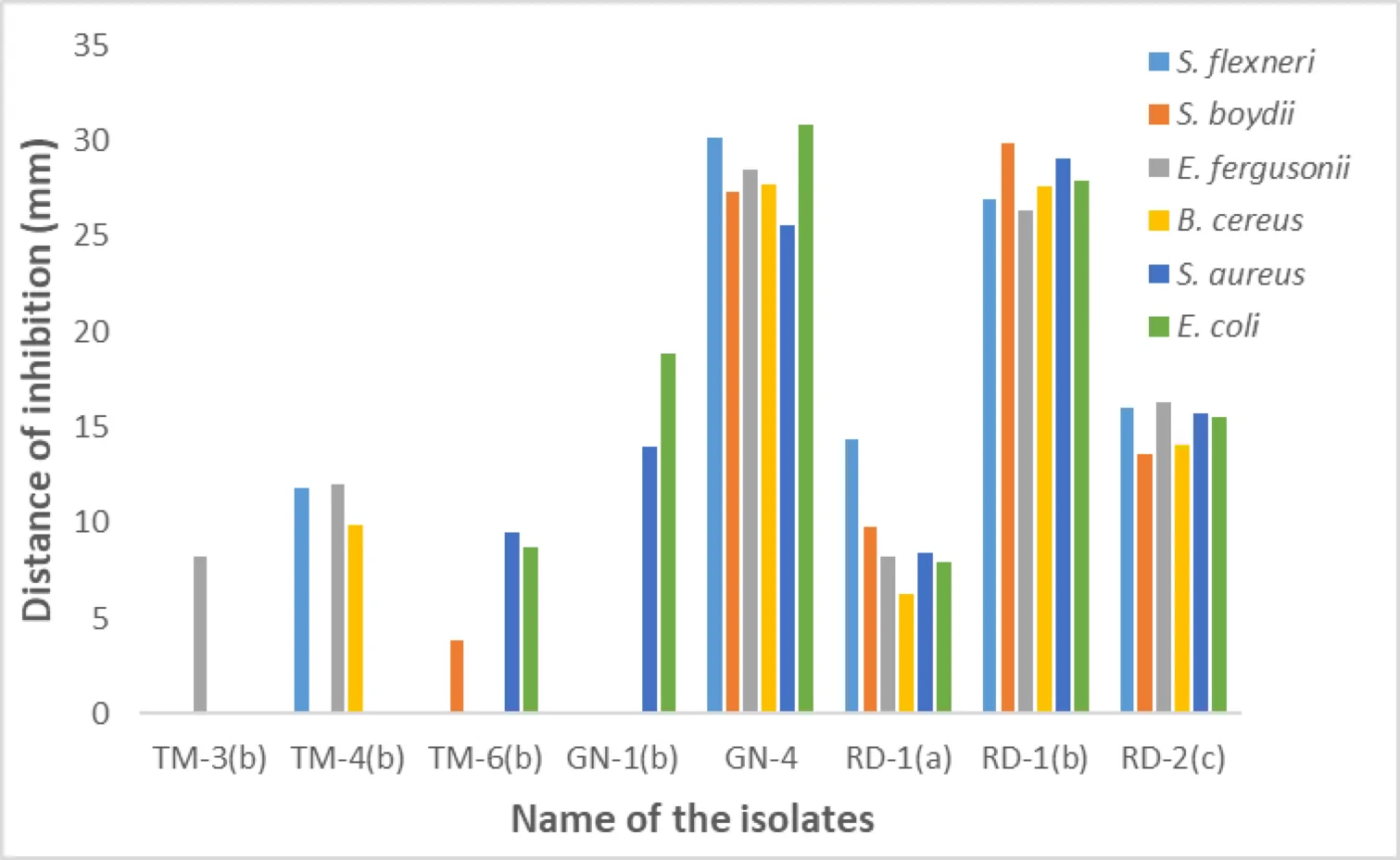
Distance of inhibition (DOI) of the isolates having inhibitory activity against the series of test bacteria.

Initial screening using the cross-streak method identified eight out of 24 isolates (33.33%) with detectable antibacterial activity against the tested multidrug-resistant (MDR) pathogens. These included both Gram-negative (*E. coli*, *S. flexneri*, *S. boydii*, *E. fergusonii*) and Gram-positive (*S. aureus*, *B. cereus*) bacteria **(**Fig. 4 and Fig. 5).

The distribution of antibacterial activity varied among plant sources. Among turmeric-derived isolates, 3 of 13 (23.08%) demonstrated inhibitory effects, primarily exhibiting weak to moderate activity. In contrast, 2 of 5 isolates (40%) from ginger showed activity, including one isolate (GN-4) that displayed strong and broad-spectrum inhibition against all tested pathogens, with inhibition distances exceeding 25 mm in several cases (Fig. 4 and Fig. 5).

Similarly, 3 of 6 isolates (50%) from white radish exhibited antibacterial activity, with isolate RD-1(b) demonstrating consistent and strong inhibition across all tested organisms (26–30 mm range).

### Molecular identification of bioactive endophytic isolates

Three representative bioactive isolates—RD-1(b), RD-2(c), and TM-4(b)—were subjected to 16S rRNA gene sequencing for molecular identification. Sequence similarity analysis revealed that isolate RD-1(b) shared 100% identity with *Burkholderia contaminans*, while isolates RD-2(c) and TM-4(b) exhibited 100% similarity to *Enterobacter cloacae* (subsp. *dissolvens*) (Table S1-S3). Several closely related taxa within the *Burkholderia cepacia* complex and *Enterobacter cloacae* complex also showed high sequence similarity (> 99%), indicating close phylogenetic relationships (Table S1-S3).

**Fig. 6:**
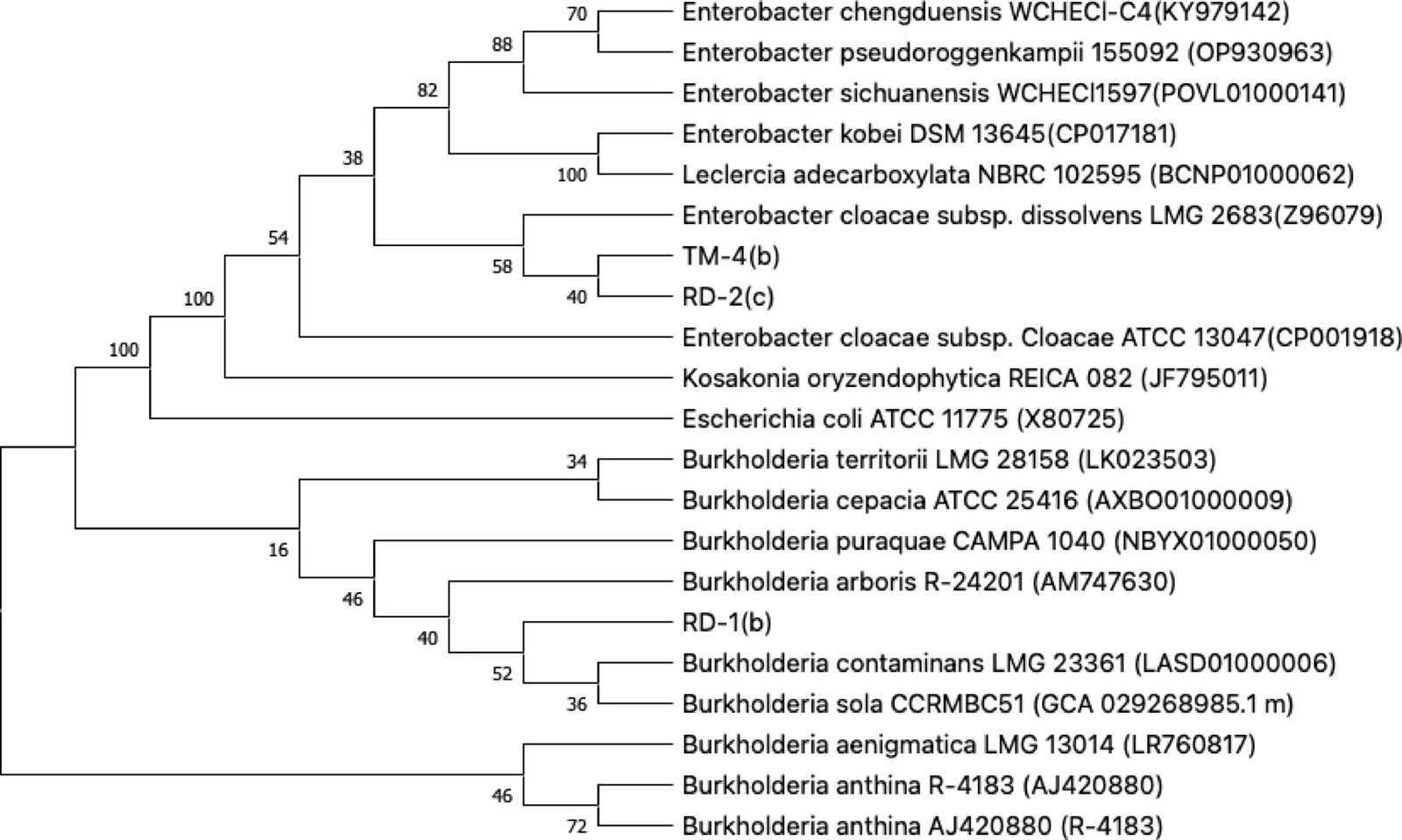
Evolutionary analysis of the TM-4(b), RD-1(b) and RD-2(c) strains of endophytic bacteria by Maximum Likelihood method. The 16S rDNA sequence of *E. coli* ATCC 11775 was used as an outgroup.

To further resolve the evolutionary relationships, phylogenetic tree was constructed using the Maximum Likelihood (ML) method (Fig. 6). In the ML tree the isolate RD-1(b) clustered within the *Burkholderia contaminans* clade, showing close association with reference strain LMG 23361, supported by high bootstrap values. Similarly, isolates RD-2(c) and TM-4(b) clustered within the *Enterobacter cloacae* subsp. *dissolvens* lineage, forming a well-supported clade with the reference strain LMG 2683 (Fig. 6).

### Physiological and biochemical characteristics of TM-4(b)and RD-1(b)

The metabolic versatility of the isolate RD-1(b), affiliated with *Burkholderia cepacia* complex and TM-4(b), affiliated with *Enterobacter cloacae* complex were assessed using six carbohydrate substrates. RD-1(b) demonstrated strong utilization of sucrose and fructose, moderate utilization of glucose, lactose, and maltose, and limited utilization of xylose (Fig. S1, Table S4). In contrast, TM-4(b) exhibited strong utilization of glucose, sucrose, and xylose, with moderate utilization of other tested carbohydrates (Fig. S2, Table S4).

Growth characteristics across different temperatures showed that RD-1(b) maintained abundant growth between 25 °C and 37 °C, with reduced growth at higher temperatures, while TM-4(b) demonstrated optimal growth at 30–37 °C and no growth above 42 °C (Table S5). These results indicate that both isolates are capable of growth at temperatures comparable to human physiological conditions.

### Antibacterial activity of the crude ethyl acetate extract

The crude ethyl acetate extract of isolate RD-1(b) retained antibacterial activity against several multidrug-resistant clinical isolates, confirming that the inhibitory activity observed during the cross-streak assay was associated with extracellular metabolites that could be recovered by solvent extraction.

The extract exhibited inhibitory activity against both Gram-positive and Gram-negative bacteria, with inhibition zones ranging from 9.4 to 20.1 mm (Table 1, Fig. S3). The greatest antibacterial activity was observed against *S. aureus* (20.1 mm), followed by *E. coli* (16.0 mm) and *B. cereus* (13.60 mm). Moderate inhibitory activity was detected against *S. flexneri* (9.85 mm) and *S. boydii* (9.4 mm), whereas no inhibition was observed against *E. fergusonii* under the experimental conditions employed. As expected, the crude extract exhibited lower antibacterial activity than the reference antibiotic kanamycin.

**Table 1:** Antibacterial activity of the crude ethyl acetate extract of isolate RD-1(b).

Test organism	Mean ZOI ± SD (in mm) of Kanamycin (30 µg/disc)	Mean ZOI ± SD (in mm) of crude ethyl acetate extract (300 µg/disc)
*Shigella flexneri*	19.10 ± 0.283	9.85 ± 0.071
*Shigella boydii*	18.90 ± 0141	9.40 ± 0.141
*Escherichia fergusonii*	15.65 ± 0.071	ND
*Bacillus cereus*	27.00 ± 0.212	13.60 ± 0.141
*Staphylococcus aureus*	29.50 ± 0.141	20.10 ± 0.141
*Escherichia coli*	29.60 ± 0.071	16.00 ± 0.00

*ND: No detectable inhibition.

### Antibiotic resistance profiles of selected bioactive isolates

**Fig. 7:**
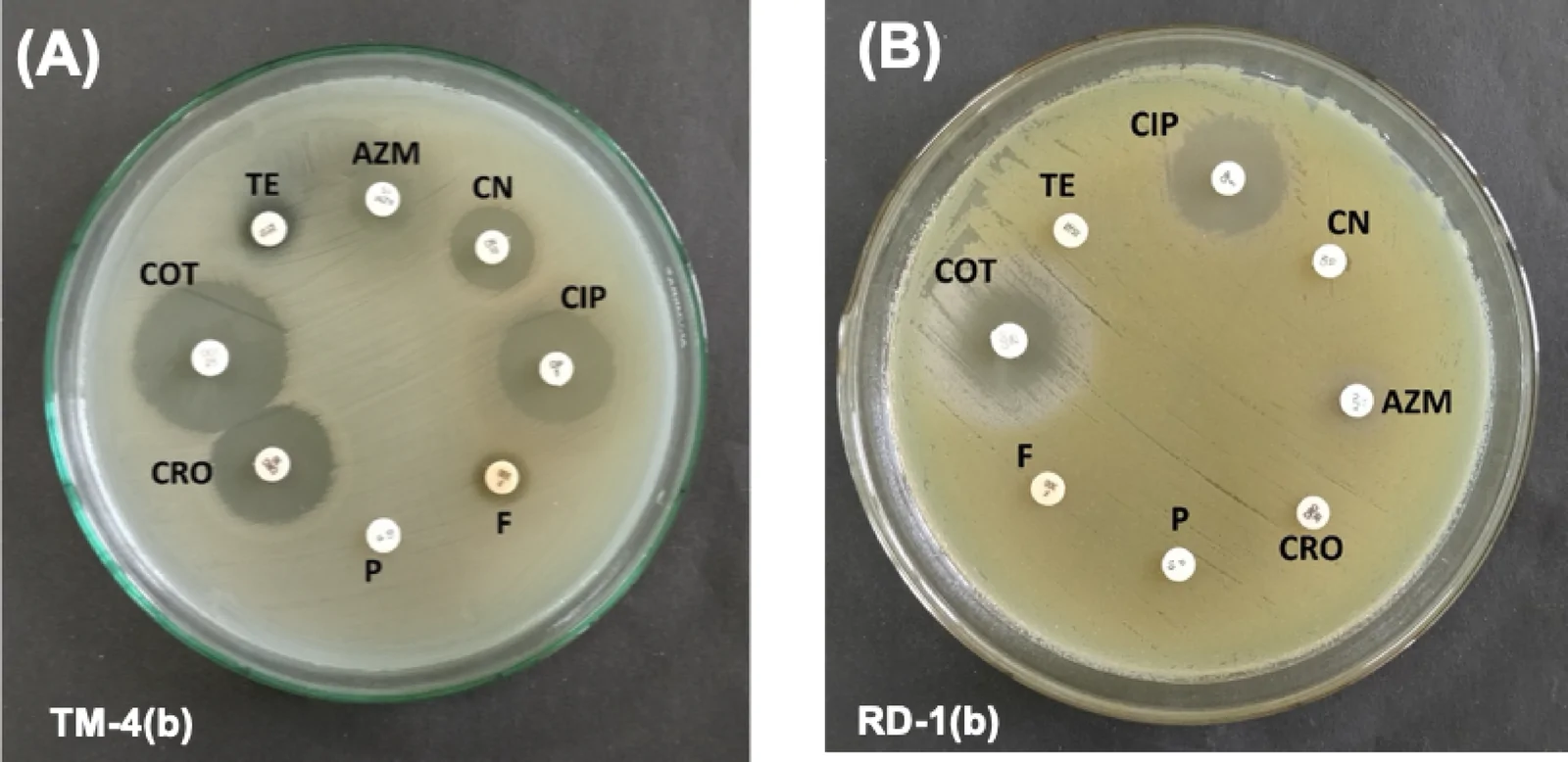
Antibiotic resistance phenotype of: (**A**) TM-4(b), affiliated with *Enterobacter cloacae* complex and (**B**) RD-1(b), affiliated with *Burkholderia cepacia complex* against different antibiotics. The following antibiotics were used: ciprofloxacin (CIP), azithromycin (AZM), penicillin G (P), gentamicin (CN), cotrimoxazole (COT), nitrofurantoin (F), tetracycline (TE), and ceftriaxone (CRO).

The antibiotic susceptibility patterns of two representative bioactive isolates, RD-1(b) and TM-4(b), were evaluated using the disk diffusion method. Both isolates exhibited resistance to multiple classes of antibiotics, indicating multidrug-resistant phenotypes **(**Table 2; Fig. 7**).**

The isolate TM-4(b), affiliated with *Enterobacter cloacae* complex, exhibited resistance to tetracycline, penicillin-G, nitrofurantoin, and azithromycin, with susceptibility observed for ciprofloxacin (ZOI: 20.9 mm), cotrimoxazole (ZOI: 25.9 mm), and ceftriaxone (ZOI: 21.2 mm), and intermediate responses to gentamicin and azithromycin **(**Table 2; Fig. 7A**).** Similarly, the isolate RD-1(b), affiliated with *Burkholderia cepacia* complex showed resistance to tetracycline, gentamicin, azithromycin, penicillin-G, ceftriaxone, and nitrofurantoin, while remaining susceptible to ciprofloxacin (ZOI: 21.4 mm) and cotrimoxazole (ZOI: 19.4 mm) **(**Table 2; Fig. 7B**)**. These results demonstrate that these endophytic bacteria isolated from edible rhizomes can harbor resistance to multiple antibiotic classes, even while exhibiting antibacterial activity against other microorganisms.

**Table 2:** Antibiotic resistance profile of TM-4(b), affiliated with* Enterobacter cloacae* complex and RD-1(b), affiliated with* Burkholderia cepacia* complex.

Isolate ID	Antibiotic Disk	Disk Potency	Mean ZOI ± SD (in mm)	Resistance type
TM-4(b)	Ciprofloxacin	5 µg	20.95 ± 0.07	S
	Tetracycline	10 µg	9.05 ± 0.21	R
	Cotrimoxazole	25 µg	25.85 ± 0.07	S
	Nitrofurantoin	300 µg	8.75 ± 0.07	R
	Penicillin-G	10 IU	0.00 ± 0.00	R
	Azithromycin	15 µg	10.85 ± 0.21	I
	Ceftriaxone	30 µg	21.05 ± 0.21	S
	Gentamycin	10 µg	14.15 ± 0.21	I
RD-1(b)	Ciprofloxacin	5 µg	21.25 ± 0.21	S
	Tetracycline	10 µg	0.00 ± 0.00	R
	Cotrimoxazole	25 µg	19.45 ± 0.07	S
	Nitrofurantoin	300 µg	0.00 ± 0.00	R
	Penicillin-G	10 IU	0.00 ± 0.00	R
	Azithromycin	15 µg	8.6 ± 0.14	R
	Ceftriaxone	30 µg	0.00 ± 0.00	R
	Gentamycin	10 µg	0.00 ± 0.00	R

### Integrated functional profile of endophytic isolates

Taken together, the characterized endophytic isolates exhibited a combination of functional traits, including (i) antibacterial activity against MDR pathogens, (ii) resistance to multiple antibiotic classes, (iii) growth at physiological temperatures, and (iv) the ability to utilize diverse carbon sources.

This integrated phenotype suggests that endophytic bacteria associated with edible rhizomes represent a functionally diverse microbial group with both antagonistic capabilities and adaptive traits relevant to survival in plant-associated and potentially host-associated environments.

## Discussion

The present study highlights the complex ecological and functional roles of endophytic bacteria associated with edible rhizomes of turmeric, ginger and white radish, revealing a microbial community that embodies both beneficial and potentially harmful characteristics. The isolation of 24 endophytic bacterial strains, with a subset (*n* = 8, 33.33%) exhibiting antibacterial activity against multidrug-resistant (MDR) pathogens, reinforces the notion that plant endospheres serve as dynamic reservoirs of bioactive microorganisms shaped by intense microbial competition^5,26^.

A notable proportion of isolates from the edible rhizomes of turmeric, ginger and white radish demonstrated antagonistic activity against clinically relevant pathogens, including *E. coli*, *E. fergusonii*,* Shigella spp.*, and *S. aureus*. Such inhibitory interactions likely reflect ecological strategies that enable endophytes to colonize and persist within plant tissues by suppressing competing microorganisms. From an applied perspective, this antagonistic potential suggests that endophytes may contribute to the natural defense mechanisms of host plants and represent a source of bioactive metabolites with therapeutic relevance.

To further validate the antibacterial potential of the endophytic isolate, crude extracellular metabolites were extracted using ethyl acetate and evaluated against representative multidrug-resistant bacteria. The retained antibacterial activity of the crude extract confirmed that the inhibitory effects observed during the cross-streak assay were attributable to diffusible extracellular metabolites rather than direct bacterial interactions. Although the crude extract exhibited lower activity than the reference antibiotic, it demonstrated broad-spectrum antibacterial activity against both Gram-positive and Gram-negative pathogens, indicating the presence of bioactive secondary metabolites. As the crude extract was not purified, the observed activity likely resulted from a mixture of compounds. Further bioassay-guided purification, structural characterization, and genomic analyses are required to identify the active metabolites and their biosynthetic pathways. However, the present study employed qualitative screening only, thus, further quantitative and chemical analyses are necessary to validate and characterize these antimicrobial effects.

Importantly, 16 S rDNA sequence analysis indicated that the bioactive endophytic isolate RD-1(b) (from radish) showed highest similarity to *Burkholderia contaminans*, whereas isolates TM-4(b) (from turmeric) and RD-2(c) (from radish) were affiliated with *Enterobacter cloacae*. Members of the *Burkholderia cepacia* complex (BCC), including *B. contaminans*, are well known for their extensive biosynthetic potential^27,28^. Genomic studies have revealed that *Burkholderia* species harbor a large repertoire of biosynthetic gene clusters, including nonribosomal peptide synthetases (NRPS), polyketide synthases (PKS), and siderophore-associated pathways, which underpin the production of diverse antimicrobial compounds^27^. These metabolites—such as pyrrolnitrin, phenazines, and lipopeptides—contribute to microbial antagonism and ecological competitiveness within plant tissues^28–31^. The broad-spectrum antibacterial activity observed in isolate RD-1(b) is therefore consistent with the established metabolic capabilities of *Burkholderia* endophytes.

Similarly, endophytic *Enterobacter hormaechei* has been shown to produce diverse bioactive compounds, including metabolites with cytotoxic and anticancer activity, highlighting its capacity as a reservoir of pharmacologically relevant molecules^32^. Biologically synthesized silver nanoparticles (AgNPs) using *E. hormaechei* demonstrate enhanced antimicrobial activity against both pathogenic and MDR bacteria, often exceeding the efficacy of conventional antibiotics^33^. Together, these findings strongly support the hypothesis that the antibacterial activity observed in the present isolates from turmeric, ginger and white radish is likely driven by metabolite-mediated mechanisms.

Beyond antimicrobial production, both genera exhibit important functional roles in plant–microbe interactions. *Burkholderia* endophytes have been widely reported to promote plant growth through mechanisms such as nitrogen fixation, phytohormone production (e.g., indole-3-acetic acid), phosphate solubilization, and stress tolerance enhancement^28–30^. Likewise, *Enterobacter cloacae* has been shown to establish a nutrient-transfer symbiosis with host plants, directly supplying nitrogen and improving plant growth under nutrient-limited conditions^34^. These functional attributes underscore the ecological importance of endophytes in supporting plant health and productivity.

However, the presence of *Burkholderia* and *Enterobacter* species in edible plant rhizomes of turmeric, ginger and white radish potentially raises safety concerns. Members of the *Burkholderia cepacia* complex (BCC), including *B. contaminans*, are recognized as opportunistic pathogens capable of infecting immunocompromised individuals^35^. Whole-genome analyses revealed that these organisms possess a broad repertoire of virulence-associated genes, including adhesion systems, invasion factors, secretion systems, and toxin-related genes^36,37^. Additionally, quorum sensing (QS) systems—such as CepIR and RpfFR—play a critical role in regulating virulence, biofilm formation, and environmental colonization in *Burkholderia*^37,38^. Similarly, *Enterobacter spp.* are members of the ESKAPE group of pathogens, which are globally recognized for their role in hospital-acquired infections and their ability to acquire resistance through mobile genetic elements^39,40^. This highlights the clinical relevance of detecting such organisms within food-associated microbiomes.

Importantly, the bioactive isolates identified in this study exhibited multidrug resistance phenotypes, including resistance to β-lactams, macrolides, and tetracyclines. The coexistence of antimicrobial activity and antibiotic resistance within the same isolates reflects an ecological paradox: resistance mechanisms may function as intrinsic protective strategies against self-produced or environmentally encountered antimicrobial compounds^9,41^. This dual functionality underscores the evolutionary adaptability of endophytic bacteria but also raises concerns regarding their potential role as reservoirs of AMR.

From a food safety perspective, identification of drug resistant bacteria in ginger, turmeric, and white radish which are frequently consumed raw or with minimal processing is really concerning. Unlike epiphytic microorganisms, endophytes reside within internal tissues^5^ and are not readily eliminated through washing or surface decontamination. Consequently, the ingestion of these plant materials may facilitate the transfer of viable, potentially multidrug-resistant bacteria and associated resistance genes (if any) into the human gastrointestinal tract. This route of exposure has been increasingly recognized as a contributor to the dissemination of AMR, potentially through horizontal gene transfer (HGT) within the gut microbiome^15,16,42,43^.

Despite these risks, the functional characteristics of the isolated endophytes suggest that their role should not be viewed solely through a pathogenic lens. Several isolates demonstrated the ability to grow at 37 °C and metabolize a range of carbon sources, indicating physiological adaptability to conditions resembling the human gastrointestinal environment. Such traits are often considered preliminary indicators of potential probiotic functionality, including survival under host-associated conditions and metabolic versatility^44^. Moreover, the observed antibacterial activity against enteric pathogens raises the possibility that certain endophytes may exert competitive exclusion effects within microbial communities, thereby contributing to colonization resistance.

However, the potential beneficial role of these bacteria must be interpreted with caution. The identification of isolates belonging to opportunistic pathogenic genera necessitates rigorous safety evaluation before any probiotic application can be considered. The distinction between beneficial commensals and opportunistic pathogens is often context-dependent, influenced by host immunity, microbial load, and the presence of virulence factors (Hornef^45^,. Therefore, future studies should incorporate comprehensive safety assessments, including hemolytic activity, virulence gene profiling, and in vivo host interaction models, to delineate the risk-benefit spectrum of these organisms.

The coexistence of antimicrobial activity, antibiotic resistance, and potential host adaptability within endophytic bacteria reflects a broader ecological and public health paradigm best understood through a One Health framework^46^. This perspective emphasizes the interconnectedness of plant, environmental, and human microbiomes, recognizing edible plants as both sources of beneficial microbes and potential vectors for antimicrobial resistance. Endophytic communities, in this context, represent a critical but underexplored component of the microbial continuum linking agricultural systems to human health.

Although the present study did not investigate the transmission of antimicrobial resistance determinants, the findings underscore the need for integrated strategies to minimize the dissemination of antimicrobial-resistant bacteria through the food chain. Such strategies include the implementation of Good Agricultural Practices (GAP), prudent antimicrobial use in agriculture and routine surveillance of antimicrobial-resistant bacteria associated with fresh produce.

Several limitations of this study should be acknowledged. First, the reliance on culture-dependent methods likely underestimates the diversity of endophytic communities. Second, the study only used 16S rRNA gene sequencing which cannot reliably differentiate members of the *Burkholderia cepacia* complex and *Enterobacter cloacae* complex. Third, the absence of quantitative antimicrobial assays such as MIC and MBC and metabolite characterization restricts conclusions regarding the therapeutic potential of the isolates. Fourth, identification of the most active isolate GN-4 could not be performed due to failure of extraction of high-quality genomic DNA. Fifth, the study involved isolates collected from a single geographical region of Bangladesh. Therefore, caution should be exercised when extrapolating these findings to other edible plants or regions. Sixth, the study performed only extraction of secondary metabolites and investigation of antibacterial activity of the extracts, however, no chemical characterisation was performed. Future research should integrate culture-independent approaches, multi-locus sequencing typing (MLST) and whole-genome sequencing, and metabolomic analyses to provide a more comprehensive understanding of endophytic bacterial function, presence of resistance genes, virulence factors, mobile genetic elements (MGEs), biosynthetic gene clusters and application potential. Future studies should also involve diverse agroecological zones to investigate if similar type of endophytes could be detected in those edible rhizomes.

In conclusion, this exploratory study demonstrates that endophytic bacteria from commonly consumed rhizomes possess a dual and context-dependent role, acting as both potential sources of antimicrobial activity and reservoirs of multidrug resistance. At the same time, their physiological adaptability and antagonistic properties suggest possible functional roles that may extend beyond the plant host. A balanced and evidence-based evaluation of these microorganisms is essential to harness their biotechnological potential while mitigating risks associated with antimicrobial resistance, particularly within the interconnected domains of food safety and global health.

## Supplementary Information

Below is the link to the electronic supplementary material.


Supplementary Material 1


## Data Availability

The 16S rDNA sequences of the isolates can be accessed from GenBank under the accession numbers PZ424636, PZ424637, and PZ424638 for isolates TM-4(b), RD-2(c), and RD-1(b), respectively.

## Declarations

### Declarations of generative AI and AI-assisted technologies

During the preparation of this work, the author(s) used ChatGPT to improve the readability of the article and to prepare the graphical abstract. After using this tool/service, the author(s) reviewed and edited the content as needed and take(s) full responsibility for the content of the publication.
