# Supplementary material for: Endophytic bacteria associated with turmeric, ginger and white radish exhibit dual roles as producers of antibacterial metabolites and carriers of multidrug resistance
Source: Sci Rep. 2026 Aug 31;16:27274. doi: 10.1038/s41598-026-64927-5 (PMC13529647; doi:10.1038/s41598-026-64927-5)
Supplement: Supplementary file 1 [file 41598_2026_64927_MOESM1_ESM.docx]

**Supplementary Figures and Tables**

**Figure S1:** (A)-(F) Utilization of different carbon sources by RD-1(b). The plates were incubated at 37ºC for 48 hours.

**Figure S2:** (A)-(F) Utilization of different carbon sources by TM-4(b). The plates were incubated at 37ºC for 48 hours.

Figure S3: Zone of inhibition of crude ethyl acetate extract of the strain RD-1(b) against a series of test bacteria.

**Table S1:** Sequences producing significant alignments with the partial 16S rDNA sequence of RD-1(b).

| Bacterial Strain | Accession | Pairwise identity (%) |
| --- | --- | --- |
| *Burkholderia contaminans* LMG 23361 | LASD01000006 | 100 |
| *Burkholderia sola* CCRMBC51 | GCA_029268985.1_m | 100 |
| *Burkholderia cenocepacia* LMG 16656 | JTDP01000003 | 99.86 |
| *Burkholderia paludis* MSh1 | JPGL01000004 | 99.86 |
| *Burkholderia arboris* R-24201 | AM747630 | 99.86 |
| *Burkholderia aenigmatica* LMG 13014 | LR760817 | 99.86 |
| *Burkholderia puraquae* CAMPA 1040 | NBYX01000050 | 99.78 |
| *Burkholderia territorii* LMG 28158 | LK023503 | 99.78 |
| *Burkholderia cepacia* ATCC 25416 | AXBO01000009 | 99.71 |
| *Burkholderia anthina* R-4183 | AJ420880 | 99.71 |
| *Burkholderia metallica* R-16017 | AM747632 | 99.71 |
| *Burkholderia semiarida* CCRMBC74 | GCA_029268935.1_m | 99.71 |
| *Burkholderia seminalis* R-24196 | AM747631 | 99.71 |
| *Burkholderia orbicola* TAtl-371 | MT928519 | 99.70 |
| *Burkholderia lata* 383 | CP000150 | 99.64 |

**Table S2:** Sequences producing significant alignments with the partial 16S rDNA sequence of RD-2(c).

| Bacterial Strain | Accession | Pairwise identity (%) |
| --- | --- | --- |
| *Enterobacter cloacae* subsp.dissolvens LMG 2683 | Z96079 | 100 |
| *Enterobacter cloacae* subsp. Cloacae ATCC 13047 | CP001918 | 99.71 |
| *Enterobacter sichuanensis* WCHECl1597 | POVL01000141 | 99.27 |
| *Enterobacter kobei* DSM 13645 | CP017181 | 99.13 |
| *Enterobacter chengduensis* WCHECl-C4 | KY979142 | 99.13 |
| *Enterobacter pseudoroggenkampii* 155092 | OP930963 | 99.11 |
| *Kosakonia oryzendophytica* REICA_082 | JF795011 | 98.98 |
| *Leclercia adecarboxylata* NBRC 102595 | BCNP01000062 | 98.91 |
| *Enterobacter ludwigii* EN-119 | JTLO01000001 | 98.91 |
| *Klebsiella quasivariicola* KPN1705 | CP022823 | 98.91 |
| *Enterobacter timonensis* MT20 | FCOP01000010 | 98.91 |
| *Enterobacter vonholyi* E13 | VTUC01000023 | 98.91 |
| *Klebsiella pneumoniae* subsp. Pneumoniae DSM 30104 | AJJI01000018 | 98.84 |
| *Silvania confinis* H4N4 | OM987254 | 98.80 |
| *Enterobacter chuandaensis* 090028 | MK049966 | 98.76 |

**Table S3:** Sequences producing significant alignments with the partial 16S rDNA sequence of TM-4(b).

| Bacterial Strain | Accession | Pairwise identity (%) |
| --- | --- | --- |
| *Enterobacter cloacae* subsp.dissolvens LMG 2683 | Z96079 | 100 |
| *Enterobacter cloacae* subsp. Cloacae ATCC 13047 | CP001918 | 99.70 |
| *Enterobacter sichuanensis* WCHECl1597 | POVL01000141 | 99.27 |
| *Enterobacter kobei* DSM 13645 | CP017181 | 99.12 |
| *Enterobacter chengduensis* WCHECl-C4 | KY979142 | 99.12 |
| *Enterobacter pseudoroggenkampii* 155092 | OP930963 | 99.10 |
| *Kosakonia oryzendophytica* REICA_082 | JF795011 | 98.98 |
| *Leclercia adecarboxylata* NBRC 102595 | BCNP01000062 | 98.90 |
| *Enterobacter ludwigii* EN-119 | JTLO01000001 | 98.90 |
| *Klebsiella quasivariicola* KPN1705 | CP022823 | 98.90 |
| *Enterobacter timonensis* MT20 | FCOP01000010 | 98.90 |
| *Enterobacter vonholyi* E13 | VTUC01000023 | 98.90 |
| *Klebsiella pneumoniae* subsp. Pneumoniae DSM 30104 | AJJI01000018 | 98.83 |
| *Silvania confinis* H4N4 | OM987254 | 98.80 |
| *Enterobacter chuandaensis* 090028 | MK049966 | 98.76 |

**Table S4:** Different carbon sources used by the active isolates RD-1(b) and TM-4(b). The sign ‘+++’ indicates abundant, ‘++’ indicates moderate and ‘+’ poor growth.

| **Isolate ID** | **Carbohydrate source used** | **Observation** |
| --- | --- | --- |
| **RD-1(b)** | Fructose | +++ |
|  | Glucose | ++ |
|  | Lactose | ++ |
|  | Maltose | ++ |
|  | Sucrose | +++ |
|  | Xylose | + |
| **TM-4(b)** | Fructose | ++ |
|  | Glucose | +++ |
|  | Lactose | ++ |
|  | Maltose | ++ |
|  | Sucrose | +++ |
|  | Xylose | +++ |

**Table S5:** Growth of the isolate RD-1(b) and TM-4(b) at different temperature (°C).

| **Isolate ID** | **Temperature used (°C)** | **Observation** |
| --- | --- | --- |
| **RD-1(b)** | 25 | +++ |
|  | 30 | +++ |
|  | 37 | +++ |
|  | 42 | ++ |
|  | 45 | + |
|  | 50 | - |
| **TM-4(b)** | 25 | ++ |
|  | 30 | +++ |
|  | 37 | +++ |
|  | 42 | ++ |
|  | 45 | - |
|  | 50 | - |
